# Projected U.S. drought extremes through the twenty-first century with vapor pressure deficit

**DOI:** 10.1038/s41598-022-12516-7

**Published:** 2022-05-21

**Authors:** Brandi L. Gamelin, Jeremy Feinstein, Jiali Wang, Julie Bessac, Eugene Yan, Veerabhadra R. Kotamarthi

**Affiliations:** 1 Environmental Science Division, Argonne National Laboratory, Argonne, IL USA; 2 Mathematics and Computer Science Division, Argonne National Laboratory, Argonne, IL USA

**Keywords:** Climate change, Climate and Earth system modelling, Projection and prediction

## Abstract

Global warming is expected to enhance drought extremes in the United States throughout the twenty-first century. Projecting these changes can be complex in regions with large variability in atmospheric and soil moisture on small spatial scales. Vapor Pressure Deficit (VPD) is a valuable measure of evaporative demand as moisture moves from the surface into the atmosphere and a dynamic measure of drought. Here, VPD is used to identify short-term drought with the Standardized VPD Drought Index (SVDI); and used to characterize future extreme droughts using grid dependent stationary and non-stationary generalized extreme value (GEV) models, and a random sampling technique is developed to quantify multimodel uncertainties. The GEV analysis was performed with projections using the Weather Research and Forecasting model, downscaled from three Global Climate Models based on the Representative Concentration Pathway 8.5 for present, mid-century and late-century. Results show the VPD based index (SVDI) accurately identifies the timing and magnitude short-term droughts, and extreme VPD is increasing across the United States and by the end of the twenty-first century. The number of days VPD is above 9 kPa increases by 10 days along California’s coastline, 30–40 days in the northwest and Midwest, and 100 days in California’s Central Valley.

## Introduction

Future drought extremes are expected to change under global warming. Current drought monitoring in the United States indicates that extreme conditions have faster onset^1^, and short-term droughts are enhanced by heat waves leading to increased drying^2,3^. Overall, under global warming, drought extremes are expected to increase throughout the twenty-first century^4–11^, ultimately affecting water resources, wildfire activities, and crop loss.

Drought occurs more frequently, with an increase in the number of hot extremes^12^, and an increase in the number of hot days and prolonged heat waves^13^, all of which have serious socioeconomic repercussions. Under extreme drought conditions, water resources, including ground water, surface water, and soil moisture, are severely diminished and can create a water emergency. This can be especially complex in regions where the impact of drought can vary on small spatial scales. Furthermore, many environments where urban, suburban, and agricultural land is interconnected (such as in the western USA), drought can exacerbate complicated water allocations.

Assessing future drought risks can be problematic due to the lack of universal drought detection methods and classifications. While drought is understood to be dry conditions persistent enough to cause crop damage or deficits in water resources, the severity or classification of deficit depends on the degree of moisture deficit and the duration of the drought event. In general, drought classifications are based on statistical measures (e.g. drought indices) to understand short-term drought, which affects agriculture and wildfire risk by drying vegetation, and/or long-term drought which affects water resources and ecological loss. Previous drought research has utilized precipitation, among other atmospheric and surface variables to classify drought (e.g. Palmer Drought Severity Index and Standardized Precipitation Index), and in relatively wet regions, precipitation deficit is indeed an important measure for drought onset. However, in regions where the climatological precipitation is modest or low (e.g. the Southwest U.S.), precipitation may not be an adequate measure of drought^14^, and more importantly, precipitation deficit is not a good indicator of extreme drought^13^. Rather, extreme drought is determined by drought intensity, often driven by temperature, the spatial extent of a specific drought, and the hydrologic demands for agriculture and human needs^15^.

One commonality among regions with drought is an increase in warm, dry conditions^5^. Drought, coupled with extreme high temperatures and low relative humidity, can increase wildfire risk^6^ and rapidly intensify crop loss. Additionally, as air temperature increases, greater moisture evaporation is released from vegetation and soil, consequently increasing drought intensity and duration^5,16^. This process is exacerbated on dry land surfaces, intensifying the positive feedback, and further increasing air temperatures^17,18^. Therefore, warming temperatures have gained recent attention and play an equally important role in understanding drought duration and intensity in the future.

An example of temperature driven drought occurs in the Southwestern (SW) United States (including California, Nevada, Arizona, New Mexico, Utah, and Colorado). In the SW, mean annual precipitation was stationary from 1895 to 2012, not showing an increasing long-term trend^19,20^. However, mean annual temperatures show an increasing long-term trend, coinciding with increasing drought occurrence for the same timeframe^21^. One state in the SW that is most notable for extreme drought is California. California is subject to severe short and long-term drought conditions, and anthropogenic warming was used to explain record low soil moisture coinciding with extreme drought^22–24^. For example, Williams et al.^25^ found that anthropogenic warming accounted for 8–27% of the observed drought anomaly in 2012–2014. California experienced post-drought relief with above average rainfall in 2017 and 2019, ameliorating the extended drought conditions from 2012 to 2016. Unfortunately, it did little to mitigate the effect of warmer-than-average temperatures and dry conditions leading up to recent drought extremes in the SW from 2020 to 2021^21^. This indicates that warmer temperatures are having a more profound effect on drought conditions than precipitation.

One measure used to understand the influence of temperature on moisture demand is Vapor Pressure Deficit (VPD). VPD is calculated with temperature and relative humidity and is the difference between the amount of water vapor the air can hold when saturated (i.e. saturation vapor pressure) and the actual amount of water vapor available (i.e. actual vapor pressure). Increasing VPD can be a consequence of drought as well as a driver of enhanced drought^21^. When VPD is high and the surface is dry, solar radiation can increase soil temperature and consequently increase the near-surface air temperature rather than evaporate water via evapotranspiration, exacerbating drought conditions. Since 1990, VPD has been increasing in the U.S.^26^ and several studies have found changes in future VPD using Global Climate models (GCMs)^27–29^. A similar analysis has not been performed with fine spatial resolution data. For this study, we approach future extremes using VPD calculated with daily maximum temperature and daily minimum relative humidity produced with multiple high-resolution dynamically downscaled climate simulations and projections.

Because VPD is a valuable measure of evaporative demand as moisture moves from the surface into the atmosphere under warming conditions, and considering saturation vapor pressure is driven by temperature, this will likely be a key measure for projecting future extremes and thus the focus of this work. This study investigates the utility of VPD, (1) in detecting short-term droughts by calculating a drought index with VPD, and by (2) assessing future VPD extremes by applying extreme value theory models to VPD.

An easily adaptable methodology is used to develop the standardized VPD drought index (SVDI) to detect short-term drought events. The new index is compared to established drought indices to evaluate its ability to detect known short-term drought events. To investigate future VPD extremes, grid dependent stationary and non-stationary generalized extreme value (GEV) models are applied, and a random sampling technique is developed to quantify multimodel uncertainties. The spatiotemporal extent of future extremes are identified, including VPD extremes and corresponding regions of increasing daily Tmax and decreasing daily minimum relative humidity throughout the twenty-first century.

## Results and discussion

### VPD based drought index

Although VPD has become increasingly useful in drought research^21,29–31^, VPD itself may be more difficult to interpret compared to established drought indices (e.g. PDSI and SPI). Leading to the question, how useful is a simplified drought index calculated with VPD in detecting short-term droughts? To evaluate VPD for drought identification, SVDI is calculated with North American Land Data Assimilation Systems (NLDAS) data (henceforward known as SVDI_NLDAS). The methodology for calculating SVDI_NLDAS is described in the Methods section. To validate the performance of SVDI, the SVDI_NLDAS data is compared to four drought indices: the Palmer Drought Severity Index (PDSI)^32^, the Standardized Precipitation Evapotranspiration Index (SPEI)^33^, the Evaporative Demand Drought Index (EDDI)^34^, and for reference, the United States Drought Monitor (USDM). Although the USDM is weekly rather than monthly, we use the USDM as a reference because it produces an extensive drought index based on, but not limited to, precipitation, soil moisture, streamflow, snow water equivalent and snowpack, crop and vegetation conditions, and reservoir and groundwater levels^35^.

Currently, there are many known drought indices. In fact, in 2016 the world meteorological organization (WMO) identified over 50 drought indices based on varying drought indicators (e.g. precipitation, temperature, ET) and classified into five categories: meteorological, hydrological, soil moisture, remote sensing, and composite^36^. Of the 20 meteorologically based indices, all incorporate precipitation^36^. With a wide assortment of drought indices to choose from, choosing an index can be difficult. While similarities exist between each drought index identified by the WMO, no individual drought index can account for all types of droughts in all types of climates. Furthermore, many meteorological drought indices require precipitation and/or several data inputs to calculate the index. In choosing an index, the WMO suggests that the simplest method is to choose one that is already being produced and freely available^36^. While this would simplify the need for a user to calculate an index, it does not, however, suggest that it would be the most suitable, nor does it allow the user to utilize an appropriate data set of their choosing. Here, we simplify this process by producing a methodology to calculate SVDI with daily VPD data, which can be calculated with any appropriate temperature and relative humidity data, and it does not require a transformation to properly fit a normal distribution like other indices (e.g. SPEI^33^).

For this work, we compare three meteorological drought indices identified by the WMO in 2016: PDSI, SPEI and USDM, and a more recent drought index based on reference evapotranspiration: EDDI. In 2009, the WMO considered the standardized precipitation index (SPI)^37^ as the standard for identifying meteorological drought^38^. However, a key caveat in using SPI in future drought prediction is the lack of impacts from temperature changes. Rather, SPEI incorporates precipitation and estimated potential evapotranspiration, which accounts for temperature^33^. On the other hand, while the PDSI does incorporate temperature, and it has been shown to be more useful in identifying long-term drought, we recognize that it may be less effective in identifying short-term droughts with timeframes less than 12 months^39^. Nonetheless, the PDSI is widely used and therefore retained for index comparison. Lastly, EDDI is utilized for a more direct comparison with SVDI. Like SVDI, EDDI does not incorporate precipitation, instead, it incorporates temperature, humidity, wind speed and solar radiation into its meteorology-based index^34^. Next, each index is shown during previously identified short term drought events.

**Figure 1:**
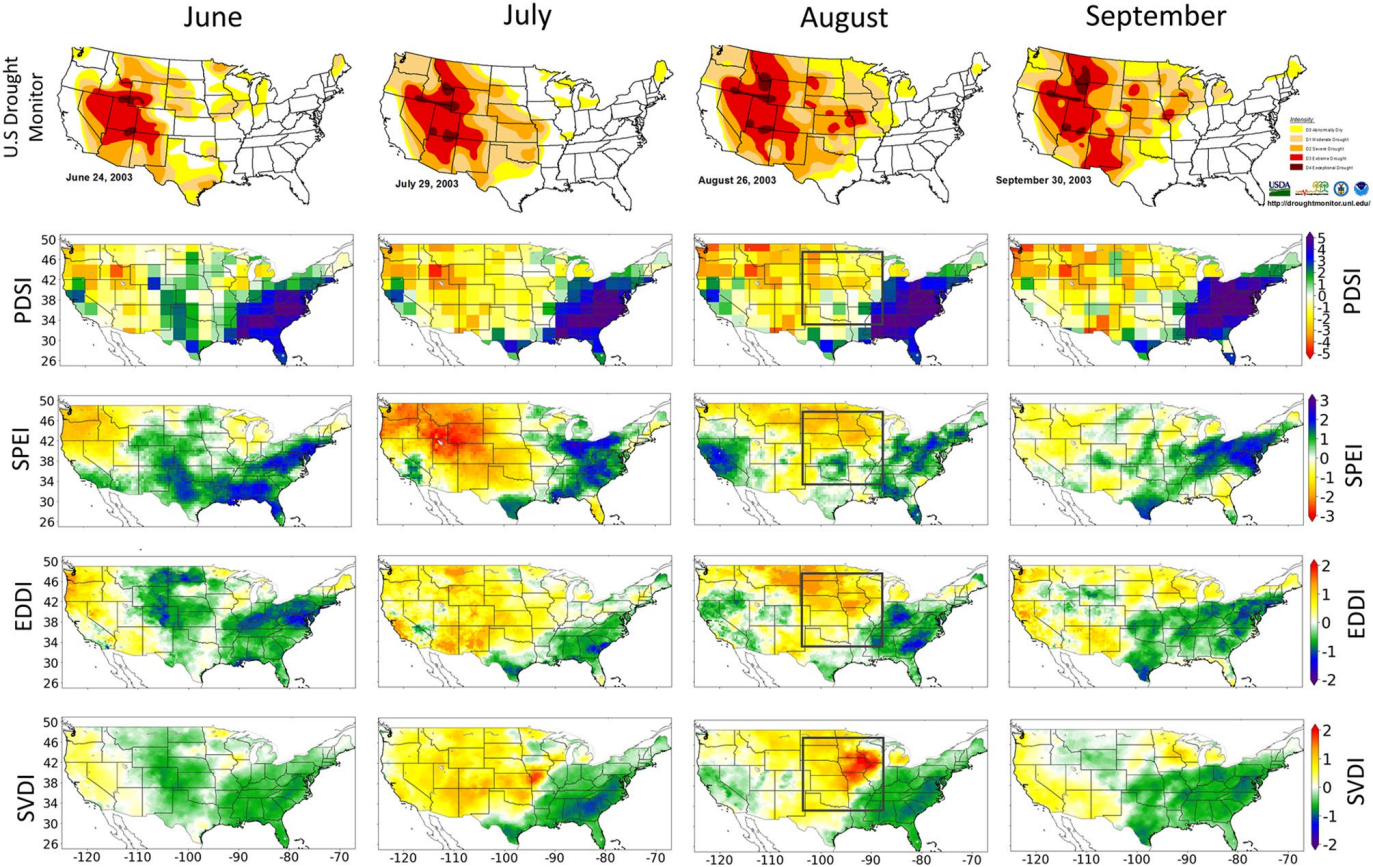
June, July, August, and September 2003 PDSI, SPEI (1-month), EDDI, and SDVI_NLDAS (SVDI). The black box represents a Flash Drought area from July 1–September 2, 2003. The USDM index is a weekly index, and dates represent the week ending that date. The SVDI index is a daily index, and the monthly value is averaged for each month. The EDDI index is averaged on the last day of each month for the previous 30 days. The SVDI, PDSI, SPEI, and EDDI plots were generated using the Matplotlib^41^ library for the Python programming language (https://matplotlib.org/). The USDM maps are courtesy of NDMC-UNL and were accessed from https://droughtmonitor.unl.edu/NADM/Maps.aspx. The USDM is jointly produced by the National Drought Mitigation Center (NDMC) at the University of Nebraska-Lincoln(UNL), the United States Department of Agriculture, and the National Oceanic and Atmospheric Administration.

Chen et al.^40^ identified several short-term drought events, known as Flash Droughts, from 2000 to 2017 using US Drought Monitor data. Flash Droughts are generally identified based on rapid intensification^3^ and short duration. Figure 1 shows the USDM, PDSI, SPEI, EDDI, and SVDI_NLDAS from June to September in 2003. The 2003 event has a 2-month rapid onset and short duration, common in a Flash Drought event, and when focusing on the Flash Drought region (Fig. 1 black box in the August map), the U.S. drought monitor maps show drying conditions rapidly intensify between July and August and diminish in September. Over the same timeframe, the PDSI shows weak increases, and SVDI_NLDAS, SPEI, and EDDI show a rapid increase, peak, and rapid decrease during this timeframe. The monthly averaged daily SVDI_NLDAS accurately identifies the location and timing of the 2003 Flash Drought and is an improvement in identifying the location of peak intensity in August. Not surprisingly, EDDI is similar to SVDI_NLDAS, neither index uses precipitation as an input and both are showing a rapid intensification of drought conditions and rapid recovery, coinciding with conditions shown in the US drought Monitor. However, when comparing the production of SVDI and EDDI, SVDI requires fewer inputs and is easier to calculate.

Overall, SVDI_NLDAS captures the location, rapid onset, and duration of the 2003 Flash Drought event. Similar results were found when comparing indices during the Flash Droughts identified by Chen et al.^40^ in 2000, 2006 and 2007 and are included in the supplemental materials (Figs. S1, S2, and S3, respectively). Next, we chose four locations for focused investigation: Western Oregon (43.06° N, 123.57° W; Northwest), Northern Iowa (43.38° N, 92.73° W; Midwest), Central Alabama (32.11 N°, 86.55 W°; South), and Southern California (34.14 N°, 118.17 W°; LA) (see supplemental materials for map of locations; Fig. S4). Each location was chosen based on recent drought related impacts. For example, increasing wildfires in Oregon^42^, the increasing relationship between maximum VPD and wildfire area in California^43^, and the recent Flash Drought events in the Midwest^40^ and the South^40^. At the grid space level, each specific location was chosen to reflect the risk associated with drought. Although, testing was conducted using multiple grid spaces, rather than individual grid spaces, little difference was found using an average of multiple grid spaces vs. an individual grid space. A timeseries of SVDI using a 7-day rolling mean from 2000 to 2008 is included in the supplemental materials (Fig. S5) showing the rapid onset and rapid decrease of drought (Flash Drought) in the Midwest (2003) and South (2000) locations. Although SVDI has been effective at distinguishing short-term drought features, it has not been tested for long-term drought detection.

Here, VPD has been utilized to produce SVDI, a simplified method for drought detection. Next, we use VPD calculated with NLDAS to compare VPD calculated with modeled GCMs downscaled with WRF from 1995 to 2004. Later, we use modelled VPD data to investigate future VPD extremes with GEV analysis.

### 1995–2004 inter-model VPD and NLDAS VPD statistics

**Figure 2:**
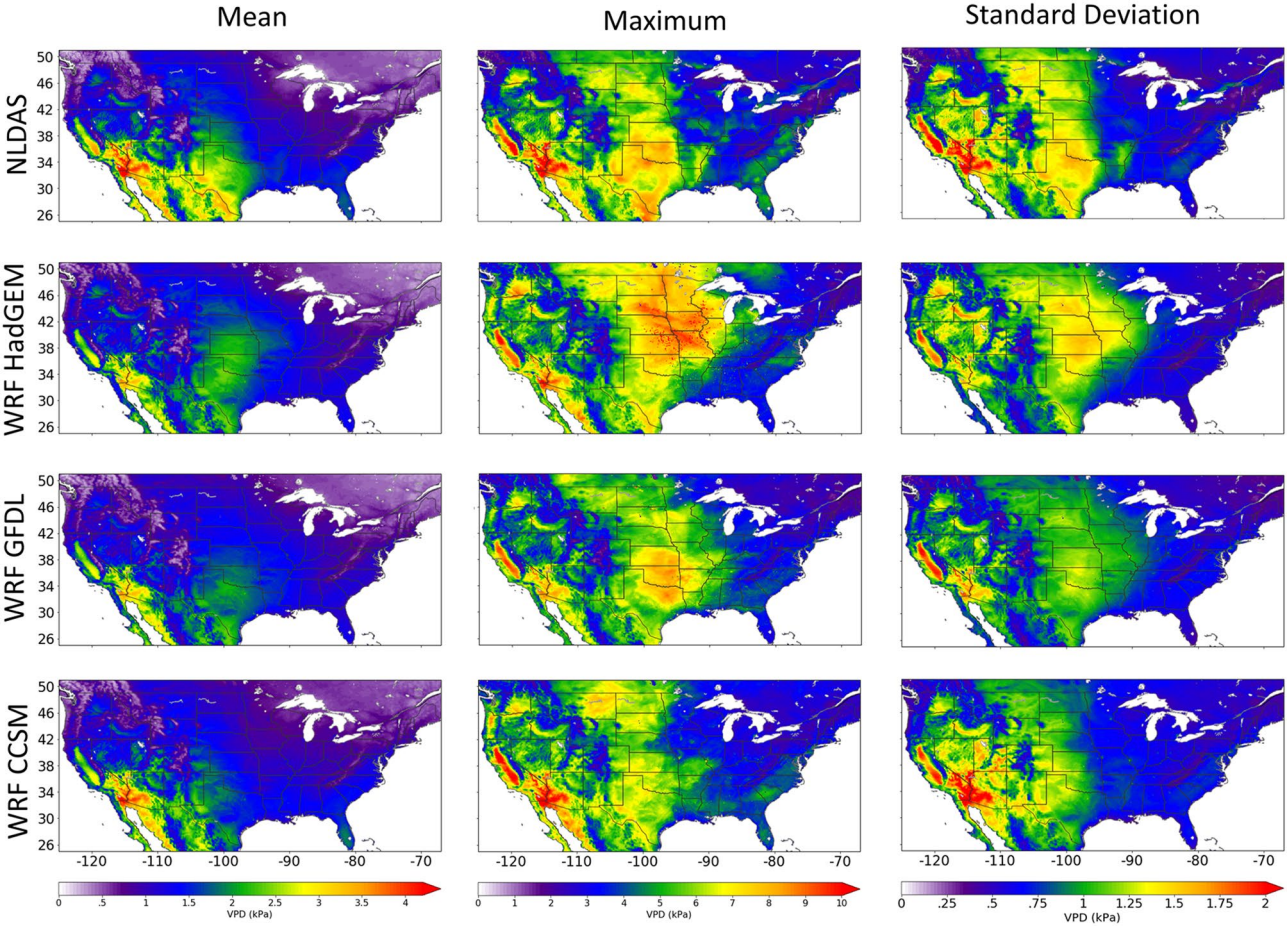
Spatial comparison of VPD statistics for 1995–2004: mean (left column), maximum (middle column), and standard deviation (right column). VPD is calculated with NLDAS (top row) and WRF HadGEM (2nd row), WRF GFDL (3rd row) and WRF CCSM (4th row). This figure was generated using the Matplotlib^41^ library for the Python programming language (https://matplotlib.org/).

Here, VPD is calculated with NLDAS data and compared to VPD calculated with data produced by three Global Climate models downscaled by the Weather Research and Forecasting (WRF) model^44^: WRF CCSM, WRF GFDL, and WRF HadGEM (See “Methods” section for model details). Figure 2 shows decadal mean VPD, decadal maximum VPD, and decadal standard deviation of VPD for the historic timeframe (1995–2004). When comparing NLDAS to the inter-model variability of the WRF models, the WRF models capture the spatial distributions of each statistical measure. However, mean VPD is underestimated by the models in the southwest and parts of the central United States. WRF HadGEM is overestimating maximum VPD and the standard deviation, especially in parts of Iowa, Missouri, and Minnesota.

### Compare inter-model VPD, temperature and relative humidity

**Figure 3:**
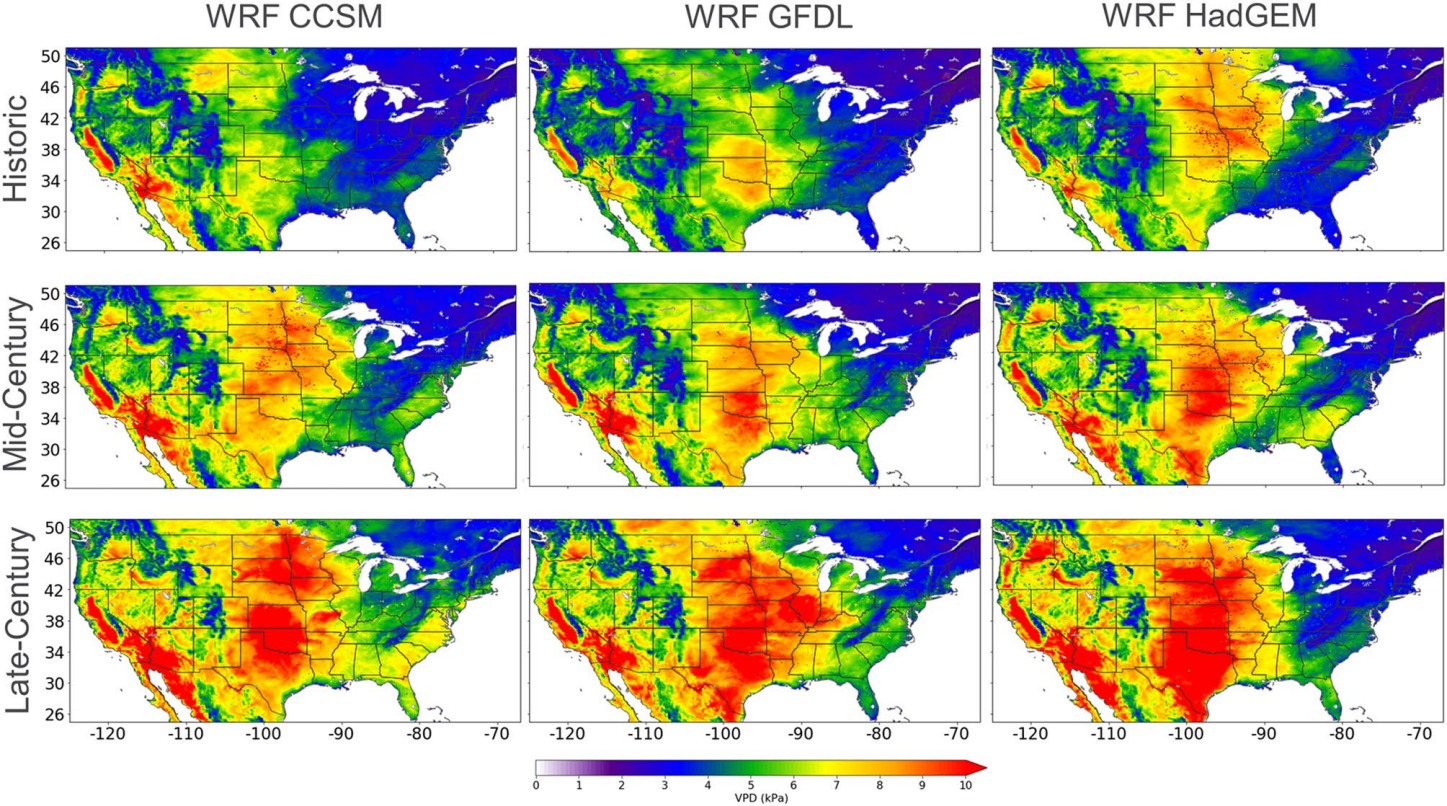
Spatial comparison of decadal averaged annual maximum VPD: 1995–2004 (Historic), 2045–2054 (Mid-Century) and 2085 -2094 (Late-Century). VPD is calculated with WRF CCSM (left column), WRF GFDL (middle column) and WRF HadGEM (right column). This figure was generated using the Matplotlib^41^ library for the Python programming language (https://matplotlib.org/).

Figure 3 shows decadal averaged annual maximum VPD for WRF CCSM, WRF GFDL, and WRF HadGEM with current climate simulations (1995–2004; historic) and future climate projections based on RCP 8.5 (2045–2054: mid-century and 2085–2094: late-century). Annual maxima generally occur during the summer months (example in “Methods” section) and the values range from 2 to 11 kPa (Fig. 3). All three models show increasing maximum VPD over time, with the largest occurring in the central United States between the historic and mid-century timeframes, and in the southern United States between the mid and late century timeframes. In California, the interior of Oregon, and the interior of Washington, maxima are consistently increasing during both timeframes. In many regions, the difference in maximum VPD between the historic and late century timeframes is greater than 3 kPa. Whether due to natural or anthropogenic climate variability, the data indicates that VPD extremes are not stationary in many regions of the United States throughout the twenty-first century.

**Figure 4:**
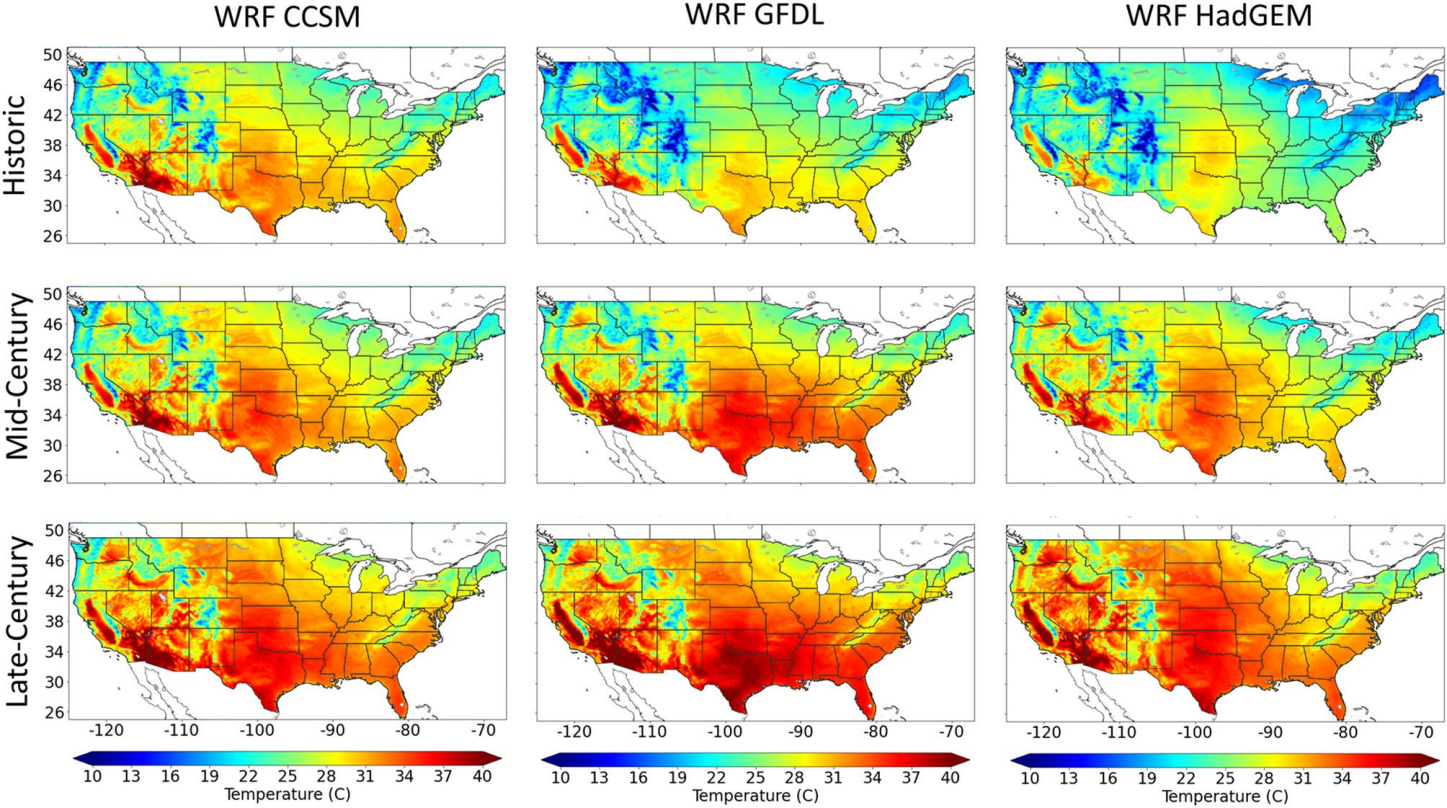
June, July, and August averaged daily maximum temperatures for each timeframe with WRF CCSM, WRF GFDL and WRF HadGEM. This figure was generated using the Matplotlib^41^ library for the Python programming language (https://matplotlib.org/).

The large increases in maximum VPD by the end of the twenty-first century may be due to increases in modeled air temperatures using RCP8.5 with respect to historic temperatures^45^. For example, Zobel et al.^46^, using the same WRF simulation, found large increases in the number of days above 95° F (35 °C) are projected to occur under the RCP8.5 scenario, especially in the central, southern, and western United States, extending the warm period 1–2 months by the end of the century. They also found the summer daytime temperature distributions increases more significantly than other seasons. Here, we are showing the summer (June, July and August; JJA) mean daily maximum temperatures (Tmax) with all three models (Fig. 4). Although WRF CCSM indicates smaller changes over time compared to WRF GFDL and WRF HadGEM, the summer averaged Tmax is increasing in all three models throughout the United States^45^. We note that WRF CCSM model simulated warmer temperatures in the historic period compared to WRF GFDL and WRF HadGEM which may account for the reduced difference over time (Fig. 4).

Of the four focused locations described earlier, the inter-model differences in Tmax during the late-century timeframe shows the Midwest location with the most agreeability between models, a difference of only ~ 1.5 °C between models, and the South and LA locations show inter-model differences of ~ 3.0 °C. At the same time, the Northwest location is showing considerably more disagreement between models with an ~ 8.0 °C difference. However, stronger agreement among the models is shown in the percent change between the historic and late-century timeframe for summer Tmax and is included in the supplementary materials (Fig. S7).

**Figure 5:**
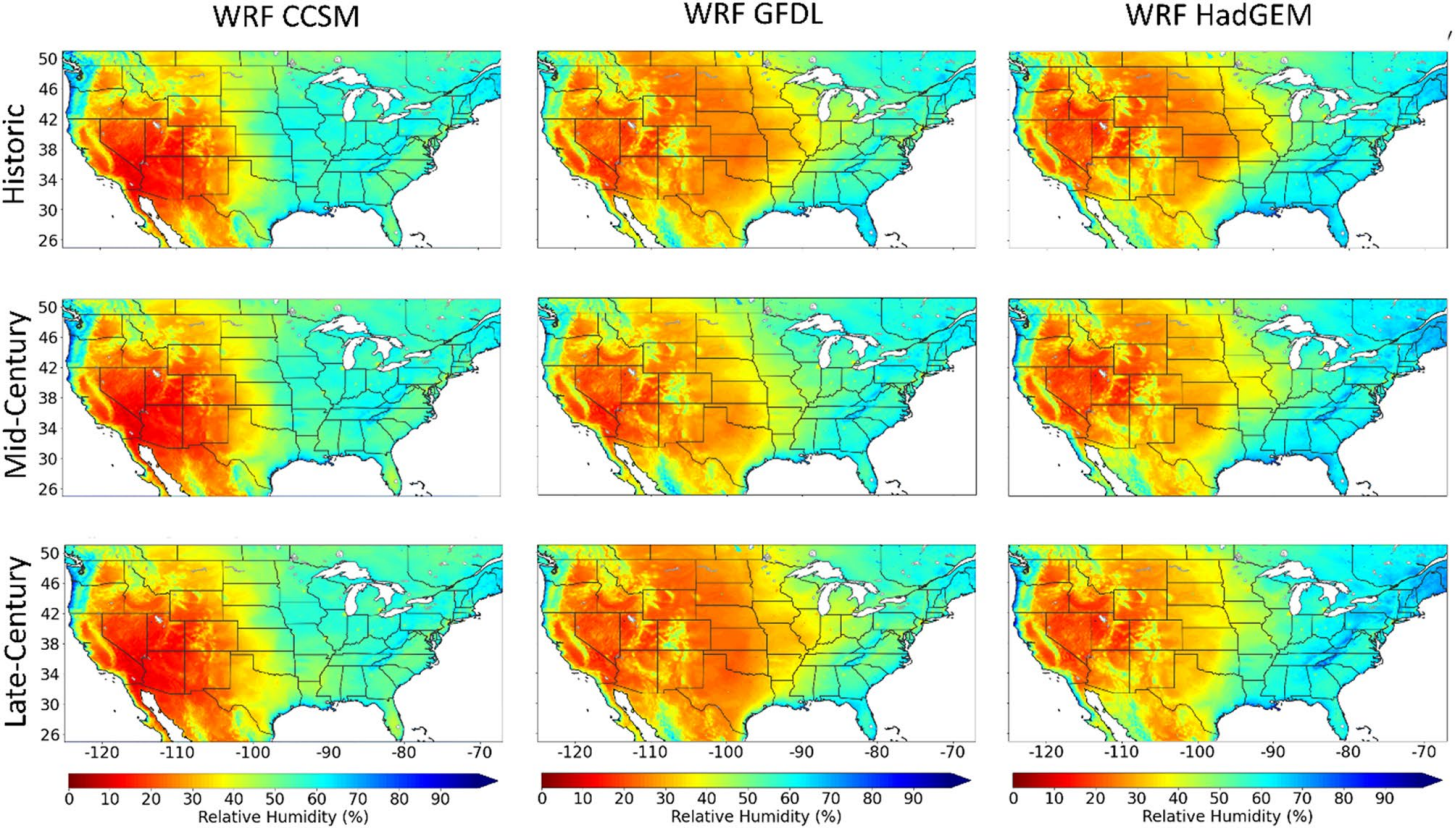
June, July, and August averaged daily minimum relative humidity for each timeframe with WRF CCSM, WRF GFDL and WRF HadGEM. This figure was generated using the Matplotlib^41^ library for the Python programming language (https://matplotlib.org/).

Although summer Tmax is expected to increase across the United States (Fig. 4) and increase VPD, changes in summer daily minimum relative humidity (RHmin) may also account for future VPD variability. Figure 5 includes summer mean RHmin with all three models and all three timeframes (Fig. 5). The percent change between the historic and late-century timeframes for summer RHmin is included in the supplemental materials (Fig. S7). When comparing future changes in Tmax and RHmin, considerably less inter-model agreement is shown with RHmin (Fig. S7). Large differences over time are found in the projected RHmin in the Midwest region (Fig. S7). For example, in the Midwest, the difference between historic and late-century RHmin sign and magnitude vary among each model: CCSM is positive, GFDL is weakly negative and HadGEM is strongly negative. This discontinuity may be responsible for VDP variability, especially in the Midwest region, resulting in the need for a multi-model ensemble to account for a range of potential VPD extremes in the future.

Overall, the projected increases in Tmax is likely driving the increases in daily VPD and may have serious repercussions on agricultural yield^47^ and hydrological resources in the western United States. Considering short-term drought related to extreme temperatures and VPD occur more frequently in the summer months, the remainder of this work focuses on JJA. Furthermore, maximum VPD is increasing disproportionally to the mean VPD (supplementary materials; Fig. S6), as such, the remainder of this work focus’ on understanding extreme VPD throughout the twenty-first century by applying generalized extreme value (GEV) models to summer maximum VPD.

### GEV analysis

Since many types of droughts are associated with high values of VPD, and no ground truth of identified droughts is available in projected climates, we further statistically quantify future extreme VPD via extreme value analysis. In particular, GEV analysis is applied to WRF CCSM, WRF GFDL, and WRF HadGEM using historic, mid-century and late-century timeframes (see “Methods” section for details). The GEV analysis provides a compact and quantitative way to assess extreme VPD that complements the above event-based analysis (that can only be performed with historical data).

A GEV statistical model is a three-parameter probability distribution model and has been used extensively to characterize extreme events such as extreme temperatures and precipitation^48–52^. In this study, we use GEV to describe the tail of the VPD distribution with the location (tail mean), scale (tail spread), and shape (tail heaviness) parameters, to calculate the probability and intensity of rare events^53^ such as a return level in a certain return period. The GEV analysis is performed on daily VPD annual maxima at each grid point.

Both stationary and non-stationary GEV models are fitted at each grid point and compared (see the Methods section for details). Grid points are identified as non-stationary via a likelihood ratio test^53^ and chosen at a 5% significance level. The locations in the United States of stationary and non-stationary grid points for each model (WRF CCSM, WRF GFDL and WRF HadGEM), are included in the supplementary materials (Fig. S8). Although inter-model stationarity varies, the majority of the grid spaces are non-stationary: ~ 56% WRF HadGEM, ~ 58% WRF GFDL, and ~ 78% WRF CCSM. However, because of widespread projected increases in temperature throughout the twenty-first century, a larger percentage of non-stationary points were expected, especially where temperatures are known to be increasing and relative humidity decreasing^29^. One explanation for grid points where temperatures are projected to increase, and yet the grid point is stationary, is increasing relative humidity, which may reduce the effect of increasing temperatures in VPD (Fig. 5 and S7). Lastly, multimodel ensembles are used to quantify uncertainty in the projections by providing multiple estimates of future return periods (see “Methods” section).

### Estimating multi-model ensemble uncertainty

**Figure 6:**
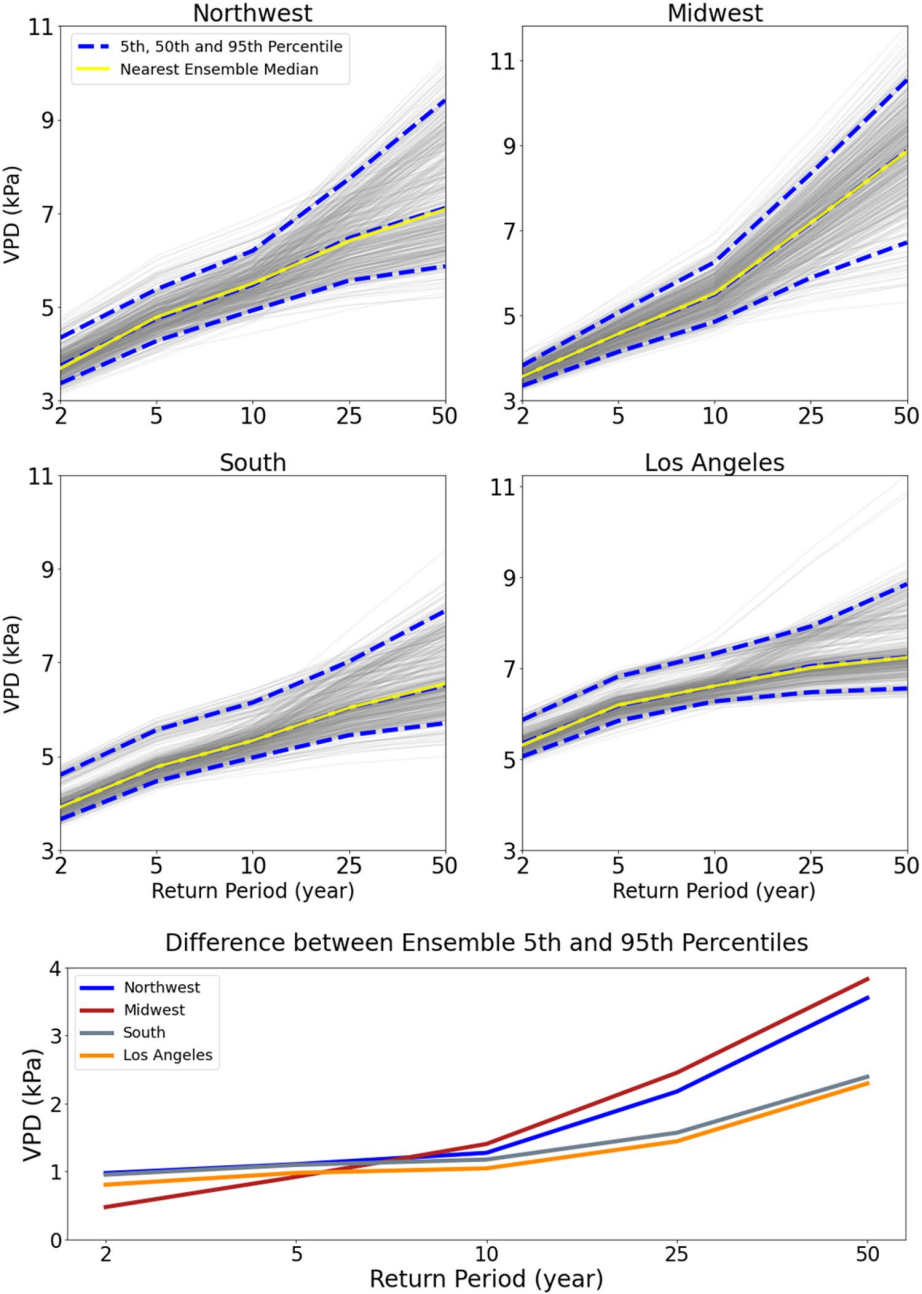
Ensemble return periods for the Northwest, Midwest, South and Los Angeles locations. Return periods for each sampled GEV fit is represented with grey lines, and the 5th, 50th, and 95th percentile of the model ensemble is represented with dashed blue lines. The yellow line is the nearest model to the ensemble median. The difference in ensemble 5th and 95th percentiles for each location are shown in the bottom graph.

We fit GEV models to annual maxima of daily VPD and identify 5 return periods of interest (2, 5, 10, 25 and 50-year), and the associated return levels are computed with the fitted models. Figure 6 shows the return levels for the four focused locations, based on 500 iterations of resampling from the three WRF models and across three modeled decades, taking place over 90 years. Of the 4 locations, the smallest uncertainty occurs in the 2–10-year return levels, generally less than 1.5 kPa difference between the 5th and 95th percentile in the resampled data; the uncertainty gets larger in the 25–50-year return levels. A map of the United States showing the difference between the 95th and 5th percentile is included in the supplemental materials (Fig. S9). Larger uncertainty is identified in the central United States, Northwest, coastal Southern California, and California’s southern Central Valley with more than 3 kPa (25-year) and 4 kPa (50-year) differences. Surprisingly, the central and northern region of the Central Valley in California has lower uncertainty compared to the southern Central Valley in the 25 and 50-year return levels (Supplemental Fig. S9). The Central Valley has shown consistent VPD increases over time throughout this study. Overall, ensemble model uncertainty increases with time, and while we have 30 years of simulated data, pooled from three timeframes^54^, larger uncertainty in the 50-year return levels is likely due to the length of the data set (see “Methods” section).

### Future VPD extremes in the United States

**Figure 7:**
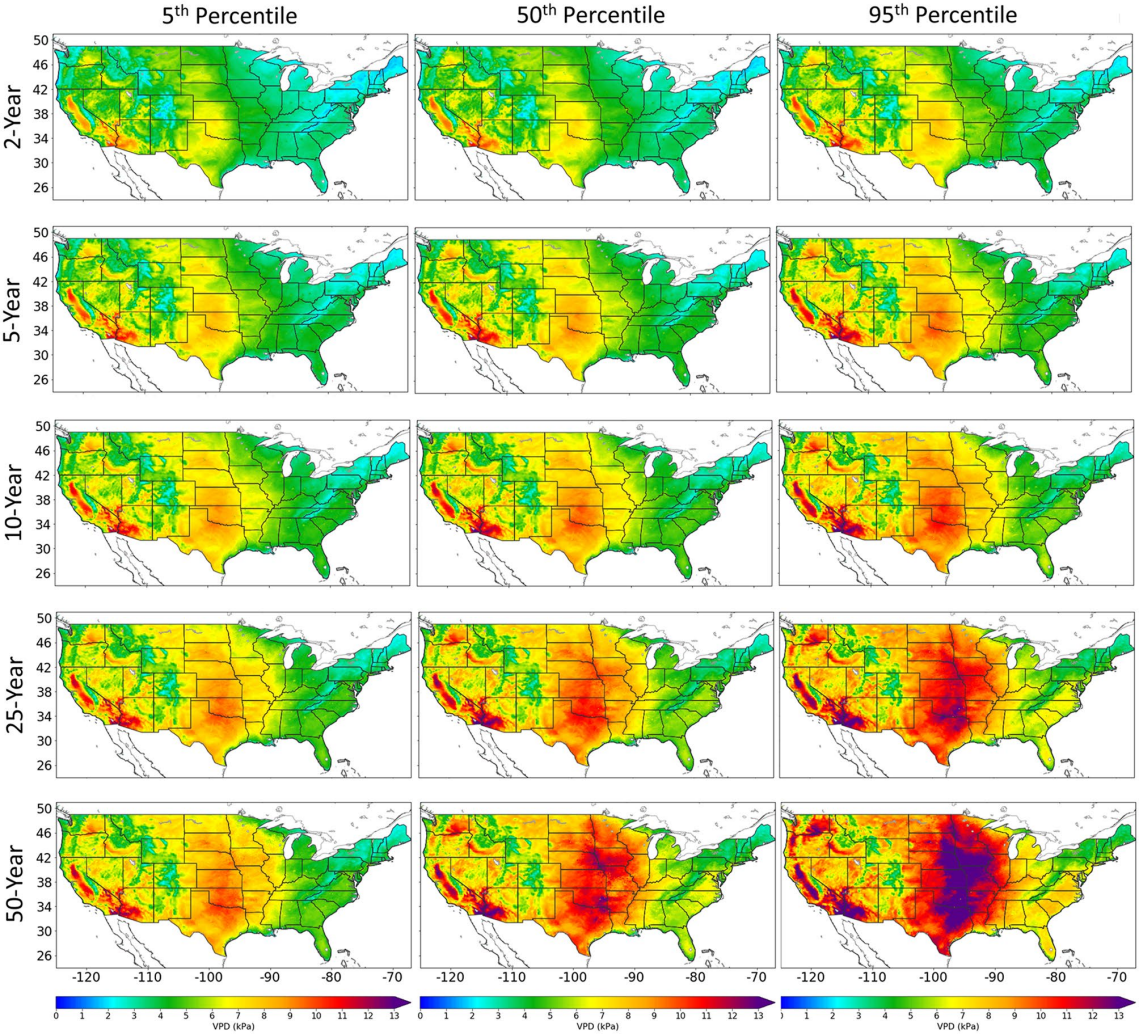
The 2, 5, 10, 25 and 50-year return periods in the United States for the 5th, median (50th), and 95th percentile of the sampled model ensemble. This figure was generated using the Matplotlib^41^ library for the Python programming language (https://matplotlib.org/).

Here we use the fitted GEV models to estimate return periods as a means of understanding the magnitude of future extreme events. Figure 7 includes the 2, 5, 10, 25 and 50-year return periods in the United States, showing the 5th, median, and 95th percentile of the sampled model ensemble. Through the return periods we observe different spatial structures depending on the degree of the severity of potential drought.

While lower uncertainty in the 50-year return periods is found in many regions of the western and eastern United States, here we focus on regions of larger uncertainty. The largest area of uncertainty is found in the Central United States. In this region, the inter-model inconsistencies in the changes in RHmin may account for the larger ensemble model uncertainty in the 25 and 50-year return periods (Fig. S9). However, the values in the lower end of the ensemble, the 5th percentile, are consistently high and the VPD return values are approaching values also found in the desert regions of the Southwest (Fig. 7). On the other hand, at the higher end of the ensemble, the 95th percentile, the central U.S. has extremely high VPD return values, greater than 13 kPa in some areas. A VPD value of 13 kPa represents a very hot, dry environment. For example, a value of ~ 13 kPa can equate to a relative humidity of 10% and an air temperature of 53.2 °C (127.76° Fahrenheit). This is an extremely arid environment. Conversely, there are few return values of 13 kPa or higher in the 5th percentile 50-year return periods, rather, the maximum return values are between 10 and 11 kPa. Furthermore, when analyzing the ensemble median 50-year return periods, although the ensemble median is showing return values of 13 kPa or greater in parts of Texas, Oklahoma, Missouri, Kansas, Iowa, Nebraska, and South Dakota, most return values are 9 kPa and greater. In this situation, a VPD value of ~ 9 kPa can equate to a relative humidity of 10% and an air temperature 46.0 °C (114.8° Fahrenheit).

To better understand VPD return values in context, we compared locations with VPD values greater than 9 kPa with all three models for the historic and late-century timeframes. Overall, four regions in the United States have VPD values greater than 9 kPa in all three models. Including, the southern and central Midwest region, the southwest desert region (including portions of coastal southern California), California’s Central Valley, and the interior of northern Oregon and southern Washington (i.e. the Northwest). The late-century model has simulated an increase of more than 10 days along coastal central California, 30–40 days in the Northwest and Midwest (centered in Northern Texas and Oklahoma). In California’s Central Valley, which is currently a valuable region for agriculture in California, the number of days above 9 kPa is increasing between 70 and 100 days, compared to the historic timeframe. We recall that the northern and middle region of the Central Valley has shown lower multi-model ensemble uncertainty in this region. The impact of extreme VPD may contribute to rapid intensification of drought conditions, subsequently leading to crop loss, enhanced wildfire risk, and ultimately impact costly hydrological resources.

## Summary and conclusions

In this study we assess the utility of VPD, (1) in detecting short-term droughts by calculating a drought index with VPD based on historical event evaluation, and (2) by assessing statistically, future VPD extremes that can lead to drought in the United States by applying extreme value theory models to VPD calculated with WRF simulations driven by three global climate models.

The new VPD driven drought index (SVDI) represents an uncomplicated methodology to identify droughts. SVDI was calculated with NLDAS and compared to PDSI, SPEI, and EDDI during a short-term “Flash Drought” event in central United States in 2003. This region was chosen due to the high frequency of Flash Drought events^55^, and the SVDI captures the rapid onset in the central Midwest in July 2003 and the rapid recovery. Similar results for the 2000, 2006 and 2007 summer Flash Drought events are shown in the supplementary materials: Figs. S1, S1 and S3, respectively.

The primary advantage of using a daily index, rather than monthly indices like PDSI and SPEI, is earlier drought detection. Moreover, when comparing SVDI against daily indices (e.g. EDDI) or weekly indices (e.g. US Drought Monitor Index), another advantage of SVDI is the simplistic nature of calculating it with any appropriate temperature and relative humidity data set. Furthermore, daily indices derive more precise statistics of the drought characteristics such as duration or detailed onset characteristics. Although SVDI has effectively distinguished short-term drought features, the future assessment of SVDI to capture long-term drought features would be necessary.

To assess future VPD extremes, a GEV distribution was fit to WRF CCSM, WRF GFDL, and WRF HadGEM. A random sampling technique was applied to all three models to produce a multi-model ensemble and characterize ensemble uncertainty in return period estimates. Ensemble uncertainty is relatively low in the 2 and 5-year return periods. However, the return values in the 50-year ensemble median indicates a large region of high VPD in multiple locations. For example, VPD values greater than 9 kPa are found throughout the central United States. This increase is likely driven by the increasing temperatures shown in the inter-model agreement, rather than changes in relative humidity due to the inter-model disagreement throughout the central U.S. Additional locations with VPD values greater than 9 kPa include the Central Valley in California, and parts of the interior of Oregon and Washington.

We assessed the results of the median 50-year ensemble return values with the individual models and found model agreement in several locations where the 50-year return values were occurring at a higher rate in the late-century timeframe compared to the historic timeframe. Using a VPD threshold of 9 kPa, along the central California coastline the individual models show an increase of more than 10 days above 9 kPa in the late-century timeframe. In the Northwest and Midwest (centered in Northern Texas and Oklahoma) regions, the number of days above 9 kPa increased by 30–40 days in the late century. Another region of great concern is California’s Central Valley, and although this area has significant water resource deficits, it is currently a valuable region for agriculture in California. The number of days VPD is above 9 kPa increases by 70–100 days in the late-century compared to the historic timeframe, and this area has high agreements among multimodel ensemble members in the northern and middle Central Valley. While this work presents the future statistical characteristics of extreme moisture deficit, the significance of adding an additional 100 days of extremely high evaporative demand on a region with complicated regional water allocations combined with serious hydrologic deficits is of great concern. Furthermore, increasing the number of days above the 50-year median ensemble return values during the late century will have serious implications for crop loss and enhanced wildfire risk in each of the identified regions.

The overall utility of VPD in drought detection and projecting VPD extremes has shown VPD to be an effective resource. Further investigation is required to properly understand patterns of drought, and long-term drought detection with SVDI.

## Methods

### Regional climate models

The WRF model version 3.3.1^44^ was run with a horizontal resolution of 12 km over most of North America. The full model domain is shown in^56^, and the model spin-up time and parameterizations are described in detail by^57^. Because of inherent computational costs involved with running high-resolution models, the WRF model was run for three separate timeframes: 1995–2004 (historic), 2045–2054 (mid-century), and 2085–2094 (late-century). Initial and boundary conditions for WRF were supplied by three fully coupled model intercomparison project phase 5 (CMIP5)^58^ models for decadal scale simulations: (1) the Geophysical Fluid Dynamics Laboratory Earth System Model with Generalized Ocean Layer Dynamics component (GFDL)^59^, (2) the Community Climate System Model, version 4 (CCSM)^60^, and (3) the Hadley Centre Global Environment Model, version 2-Earth System (HadGEM)^61^. For this analysis, the GCMs with future scenarios are run with RCP 8.5.

Zobel et al.^57^ evaluated the model performances of WRF CCSM, WRF GFDL, and WRF HadGEM with seven surface variables and four upper atmospheric variables based on their climatology and extremes for seven subregions in the United States. Their results indicate that model skill depends on the location and variable being tested, and they found the high-resolution simulations an improvement over the GCMs used as initial and boundary conditions driving the simulations, especially for variables with high spatial and temporal variability, such as precipitation.

### Vapor pressure deficit (VPD)

Our investigations focus on maximum daily evaporative demand to understand extreme drought. For this, we used daily maximum temperature (Tmax; in Celsius) and daily minimum relative humidity (RHmin) to calculate VPD. Temperature and relative humidity were determined based on WRF 3-h output over each 12 km grid cell. This was used to calculate saturation vapor pressure (es; Eq. 1) and VPD (Eq. 2).

1 $${\text{es}} = 0.6108^{{\left( {17.27{\text{*Tmax}}/\left( {{\text{Tmax }} + 237.3} \right)} \right)}}$$

2 $${\text{VPD}} = \left( {1 - \left( {{\text{RHmin}}/100} \right)} \right){\text{* es}}$$

Daily VPD is calculated over each grid cell in the contiguous United States (CONUS) and for each timeframe separately.

### Drought indices

Data for the Palmer Drought Severity Index^31^ (PDSI) was obtained from NCAR/UCAR^39^ and the horizontal grid spacing is 2.5° × 2.5°. Negative PDSI values indicate dry/drought conditions. The Standard Precipitation Evaporation Index (SPEI) was introduced by Vincente Serrano et al.^33^, and the procedure for calculating SPEI is like that of the standardized precipitation index (SPI)^62^, however, SPEI uses the difference between precipitation and reference evapotranspiration as a measure of moisture input and evaporative demand^33^. The data for the SPEI was obtained from NCAR’s (National Center for Atmospheric Research) monthly global 0.5° gridded Climate Research Unit (CRU TS3.2) SPEI database and was accessed from https://spei.csic.es/database.html. The Evaporative Demand Drought Index (EDDI) is a new drought monitoring tool showing anomalous evaporative demand with temperature, humidity, wind, and solar radiation as inputs^34^. The EDDI data has a ~ 12 km spatial resolution and is calculated with NLDAS data^63,64^. EDDI data was accessed from https://downloads.psl.noaa.gov/Projects/EDDI/CONUS_archive/. The US drought monitor (USDM)^35^ maps are included for reference. The USDM is jointly produced by the National Drought Mitigation Center (NDMC) at the University of Nebraska-Lincoln(UNL), the United States Department of Agriculture, and the National Oceanic and Atmospheric Administration. The maps are courtesy of NDMC-UNL and were accessed from https://droughtmonitor.unl.edu/NADM/Maps.aspx.

The Standardized VPD Drought Index (SVDI) was calculated with daily VPD calculated from NLDAS data from 1990 – 2010 (NLDAS data is described below). This simplified method of drought detection was created to capture local deficits on short time scales. The daily VPD was standardized with the climatological (1990 – 2010) monthly mean and monthly standard deviation. At each time point (i.e. daily), VPD was subtracted from the monthly mean and then divided by the monthly standard deviation. This produced an index with values ranging from approximately − 3 to + 3. Contrary to the PDSI and SPEI, dry/drought conditions are positive values and SVDI drought conditions were observed with index values greater than 1. Monthly averaged SVDI data is compared to the PDSI, SPEI and EDDI.

### North American land data assimilation system-2

North American Land Data Assimilation System (NLDAS)-2 is a land modeling and assimilation system produced in an uncoupled mode with a 0.125° × 0.125° (~ 12 km) spatial gird resolution^63,64^. NLDAS land-surface forcing fields are derived from the NCEP North American Regional Reanalysis (NARR). Temperature, specific humidity, and surface pressure variables were taken from the traditional land-surface forcing fields. Specific humidity and surface pressure variables were used to calculate relative humidity, and subsequently used to calculate VPD. SVDI was calculated with NLDAS VPD and compared to drought indices listed above.

### Generalized extreme value models

We fit a GEV distribution to a 30-year sample by combining the three different timeframes to characterized extreme VPD and assess future drought intervals^54^. There are two extreme data analysis techniques used in extreme value analysis: block-maxima^65^ on which GEV are then fit, and the peak-over threshold^66^ for which Generalized Pareto distributions are appropriate. While the peak-over threshold method samples all relevant high VPD values, inherently producing a larger sample size, it does not ensure independence between events without further treatments and requires choosing a relevant threshold. To overcome these issues, we applied the block-maxima method and fitted GEV distributions to annual summer maxima. This method will ensure non-overlapping points and independence between drought events by collecting annual maxima as inputs for the GEV^67^.

### Outlier detection and treatment

**Figure 8:**
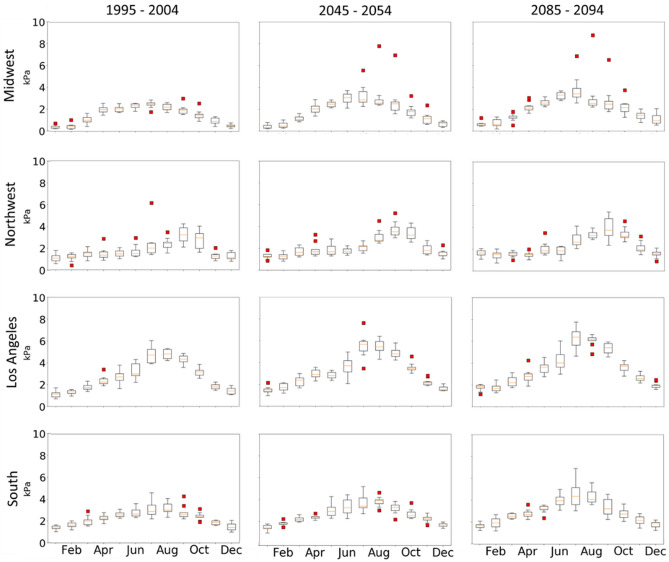
CCSM_WRF VPD boxplots of monthly maximum with outliers (red squares) for Western Oregon (43.06° N, 123.57° W; Northwest), Northern Iowa (43.38° N, 92.73° W; Midwest), Central Alabama (32.11 N°, 86.55 W°; South), and Southern California (34.14 N°, 118.17 W°; LA) for each decadal timeframe.

Because the saturation vapor pressure in VPD is dependent on temperature, an upper bound is expected at each grid point. This VPD upper bound is dependent on local Tmax and when calculating VPD, large VPD extremes were identified, especially in the Midwestern Untied States (Fig. 8). Furthermore, abnormally large VPD may produce statistically unphysical return values. To ensure meaningful return values from the GEV analysis, we have applied an outlier detection method.

The interquartile range (IQR) outlier detection method was applied to each grid point to determine a threshold. To preserve extreme values, only one VPD maximum per year is utilized to calculate IQR with the first and third quartiles (Q3 minus Q1). Outlier threshold value was determined by Q3 + 1.5*IQR. Boxplots with outliers for individual locations within each state: Los Angeles (California), South (Alabama), Midwest (Iowa) and Northwest (Oregon), are shown in Fig. 8. The outliers were calculated with the IQR method. In preparation for the GEV analysis, all June, July, and August (JJA) VPD values above the threshold were removed and the next nearest value below the threshold is retained.

### Resampling for model uncertainty

Because of large variability between global climate models, and in turn, GCMs downscaled with the regional model (WRF), it is necessary to create an ensemble model with the retained JJA maximums. To gauge the accuracy of the ensemble, a sampling technique is applied and repeated 500 times to determine a range of possible models and find the ensemble median (50th percentile), upper bound (95th percentile), and lower bound (5th percentile) for each return period.

To achieve this, three separate timeframes: 1995–2004 (historic), 2045–2054 (mid-century), and 2085–2094 (late-century) are combined at each grid point (Srivastava et al., 2021) and GEV models are applied to the sampled data. An individual sample is produced when one of the three model annual JJA maximum is randomly selected at each time point to create a timeseries for GEV analysis. Each resampled timeseries is the same size as the original data. This resampling technique is repeated 500 times to create a multi-model ensemble.

Because VPD is dependent on temperature, in a warming world it is necessary to investigate the role of non-stationarity in the data. Both stationary (where all 30 years of data are considered having the same distribution) and non-stationary GEV distributions are applied. For each iteration of resampling, a likelihood ratio test^53^ (α = 0.05) determines whether the 4-parameter, non-stationary GEV has a significant improvement in log likelihood compared to the 3-parameter, stationary GEV. The stationary GEV has 3 parameters: location (μ), scale (σ), and shape (ξ), representing the center, spread, and tail heaviness of the data extremes, respectictivly^52^. The non-stationary GEV where the location parameter is modeled as a linear function of time (year) that accounts for the gaps between the 3 10-year slices, has 4 parameters: location intercept (μ_0_), location trend (μ_1_), scale(σ), and shape(ξ). If the 4-parameter GEV is selected via likelihood ratio, it is converted to a 3-parameter GEV by localizing to the mid-century modeling period (t = 50, given that μ(t) = μ_0_ + μ_1_t) prior to quantile estimation. Therefore, our results reflect a combination of quantiles derived from stationary GEV and non-stationary GEV at t = 50.

The sampled GEV models are utilized to estimate 2, 5, 10, 25 and 50-year return periods. When considering the uncertainty of return periods, Hosking and Wallis^68^ found that the bias and variance of estimated quantiles (i.e., returns periods) with respect to their true quantities are approximately proportional to n^−1^ and the RMSE approximately proportional to n^−1/2^. A record length of n = 30 is considered reasonable and to have limited error for estimating 25-year and 50-year events. We acknowledge that the return level estimates will have greater uncertainty at higher quantiles and that the 50-year return estimates involve extrapolation of the empirical distribution.

## Supplementary Information

Supplementary Figures.
