# Supplementary material for: Projected U.S. drought extremes through the twenty-first century with vapor pressure deficit
Source: Sci Rep. 2022 May 21;12:8615. doi: 10.1038/s41598-022-12516-7 (PMC9124218; doi:10.1038/s41598-022-12516-7)
Supplement: Supplementary file 1 — Supplementary Figures. [file 41598_2022_12516_MOESM1_ESM.docx]

Supplemental materials for:

**Projected U.S. Drought Extremes Through the 21st Century with Vapor Pressure Deficit**

by Gamelin et al.


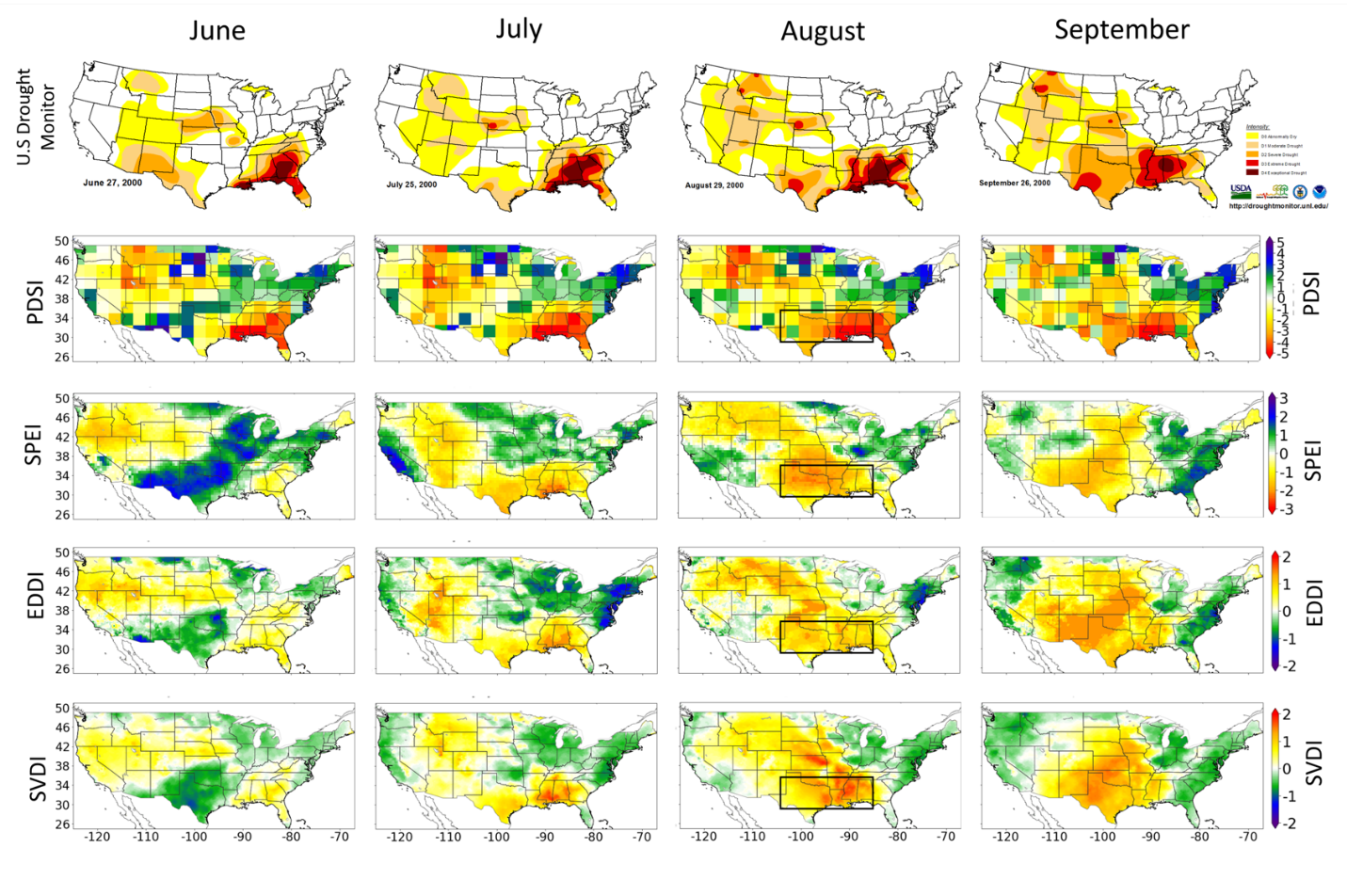


Figure S1. June, July, August, and September 2000 SDVI_NLDAS (SVDI), SPI, and PDSI. The black boxes represent the area of a Flash Drought event in 2000. The SVDI, PDSI, SPEI, and EDDI plots were generated using the Matplotlib^68^ library for the Python programming language (https://matplotlib.org/). The USDM maps are courtesy of NDMC-UNL and were accessed from <https://droughtmonitor.unl.edu/NADM/Maps.aspx>. The USDM is jointly produced by the National Drought Mitigation Center (NDMC) at the University of Nebraska-Lincoln(UNL), the United States Department of Agriculture, and the National Oceanic and Atmospheric Administration.


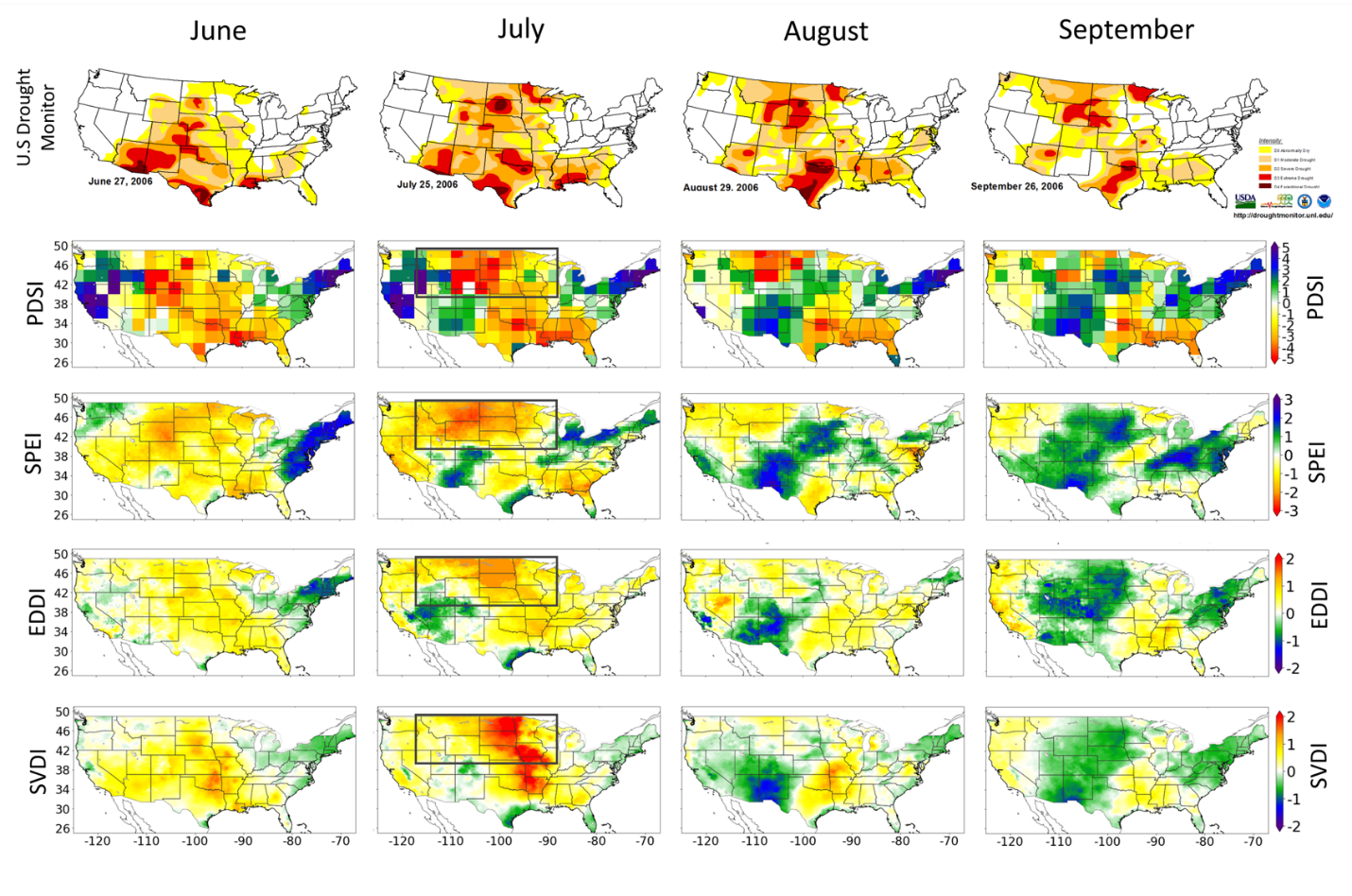


Figure S2. June, July, August, and September 2006 SDVI_NLDAS (SVDI), SPI, and PDSI. The black boxes represent the area of a Flash Drought event in 2006. The SVDI, PDSI, SPEI, and EDDI plots were generated using the Matplotlib^68^ library for the Python programming language (https://matplotlib.org/). The USDM maps are courtesy of NDMC-UNL and were accessed from <https://droughtmonitor.unl.edu/NADM/Maps.aspx>. The USDM is jointly produced by the National Drought Mitigation Center (NDMC) at the University of Nebraska-Lincoln(UNL), the United States Department of Agriculture, and the National Oceanic and Atmospheric Administration.


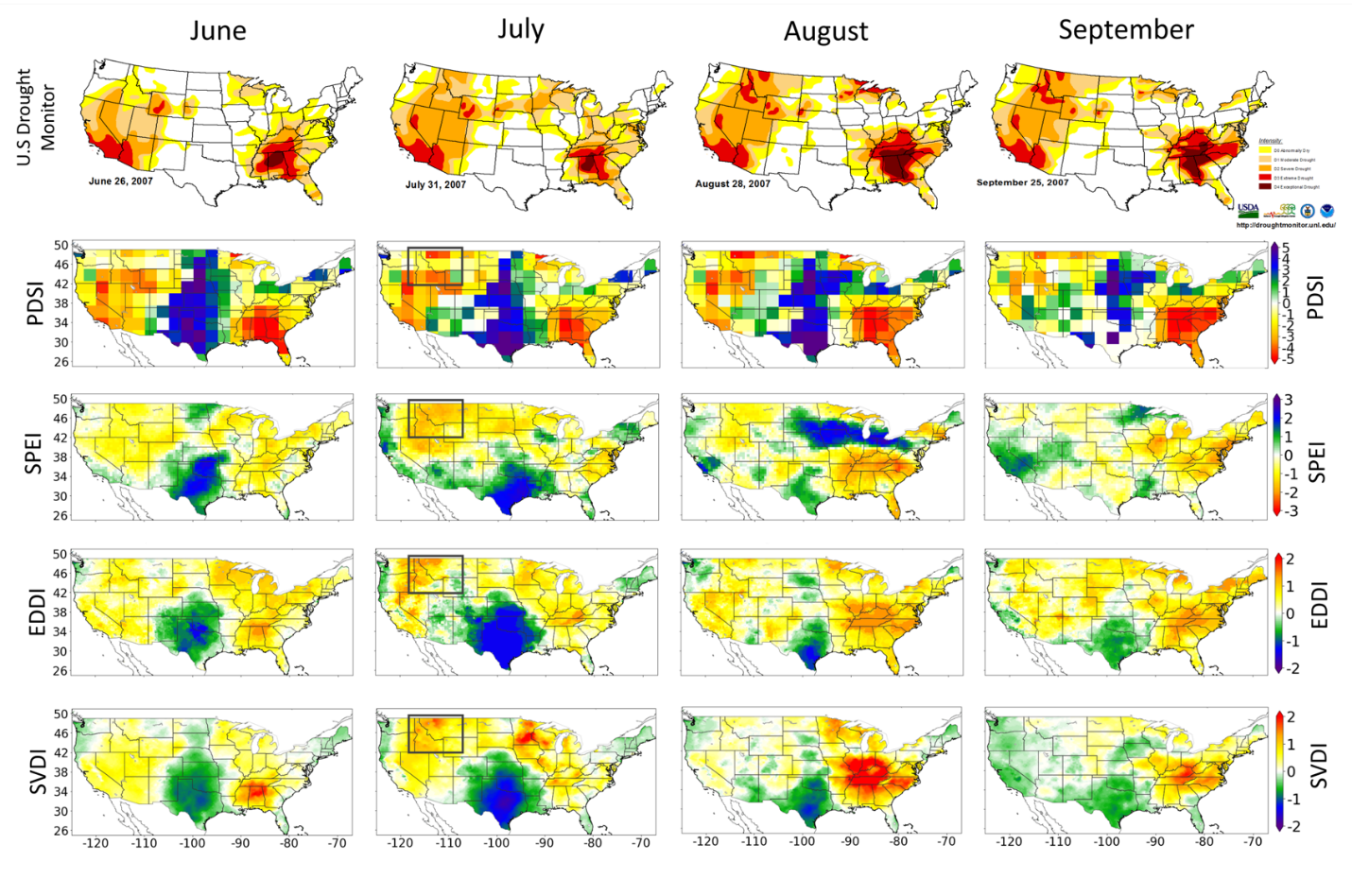


Figure S3. June, July, August, and September 2007 SDVI_NLDAS (SVDI), SPI, and PDSI. The black boxes represent the area of a Flash Drought in 2007. The SVDI, PDSI, SPEI, and EDDI plots were generated using the Matplotlib^68^ library for the Python programming language (<https://matplotlib.org/>). The USDM maps are courtesy of NDMC-UNL and were accessed from <https://droughtmonitor.unl.edu/NADM/Maps.aspx>. The USDM is jointly produced by the National Drought Mitigation Center (NDMC) at the University of Nebraska-Lincoln(UNL), the United States Department of Agriculture, and the National Oceanic and Atmospheric Administration.


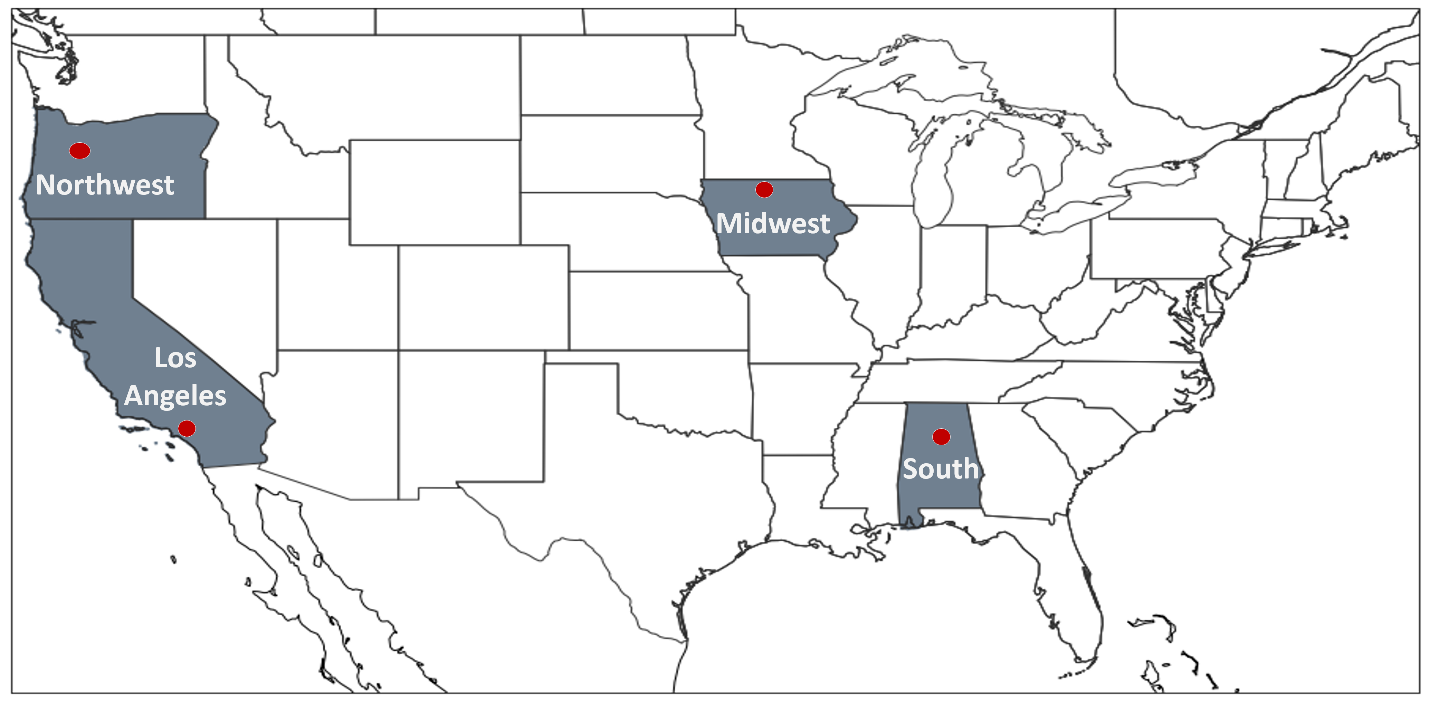


Figure S4. States (locations) of focused investigation: Oregon (43.06°N, 123.57°W; Northwest), Iowa (43.38°N, 92.73°W; Midwest), Alabama (32.11N°, 86.55W°; South), and California (34.14N°, 118.17W°; Los Angeles). This figure was generated using the Matplotlib^68^ library for the Python programming language (https://matplotlib.org/).


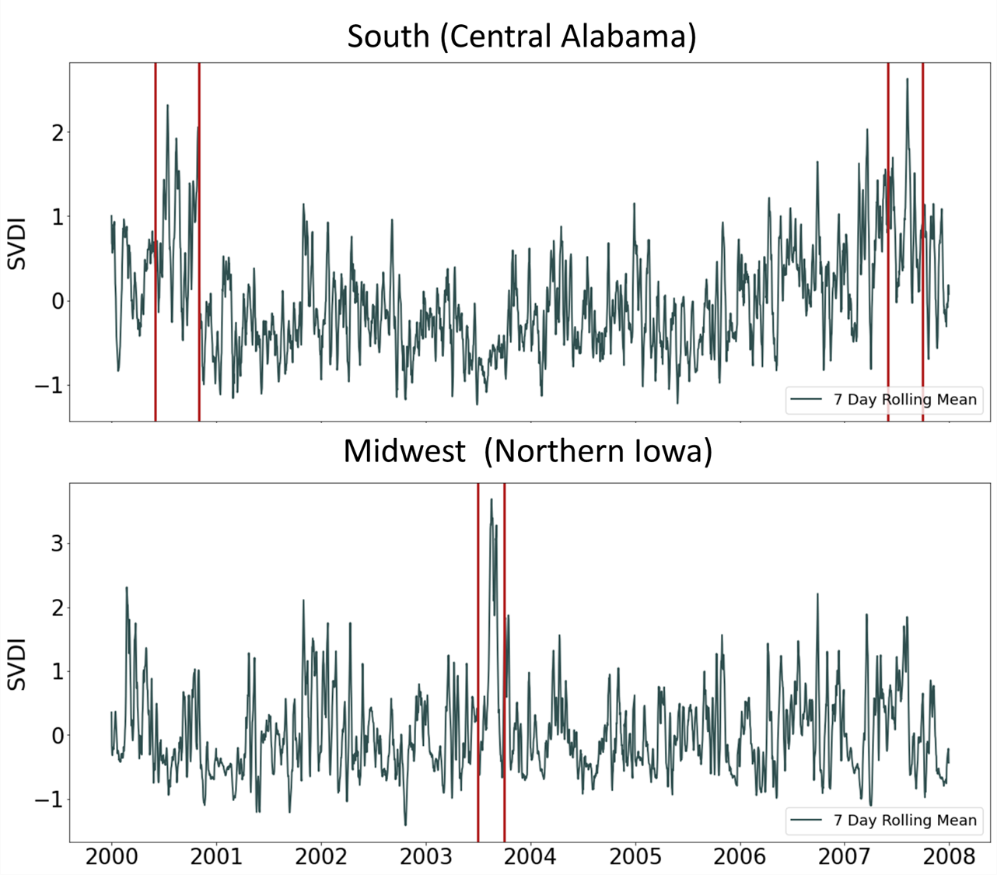


Figure S5. SVDI 7-day Rolling mean from 2000 – 2008 for two of our focused location: South and Midwest. Vertical red lines indicate time frames for Flash Drought Events investigated in this study (2000 and 2003) and a drought event in the South in the summer of 2007.


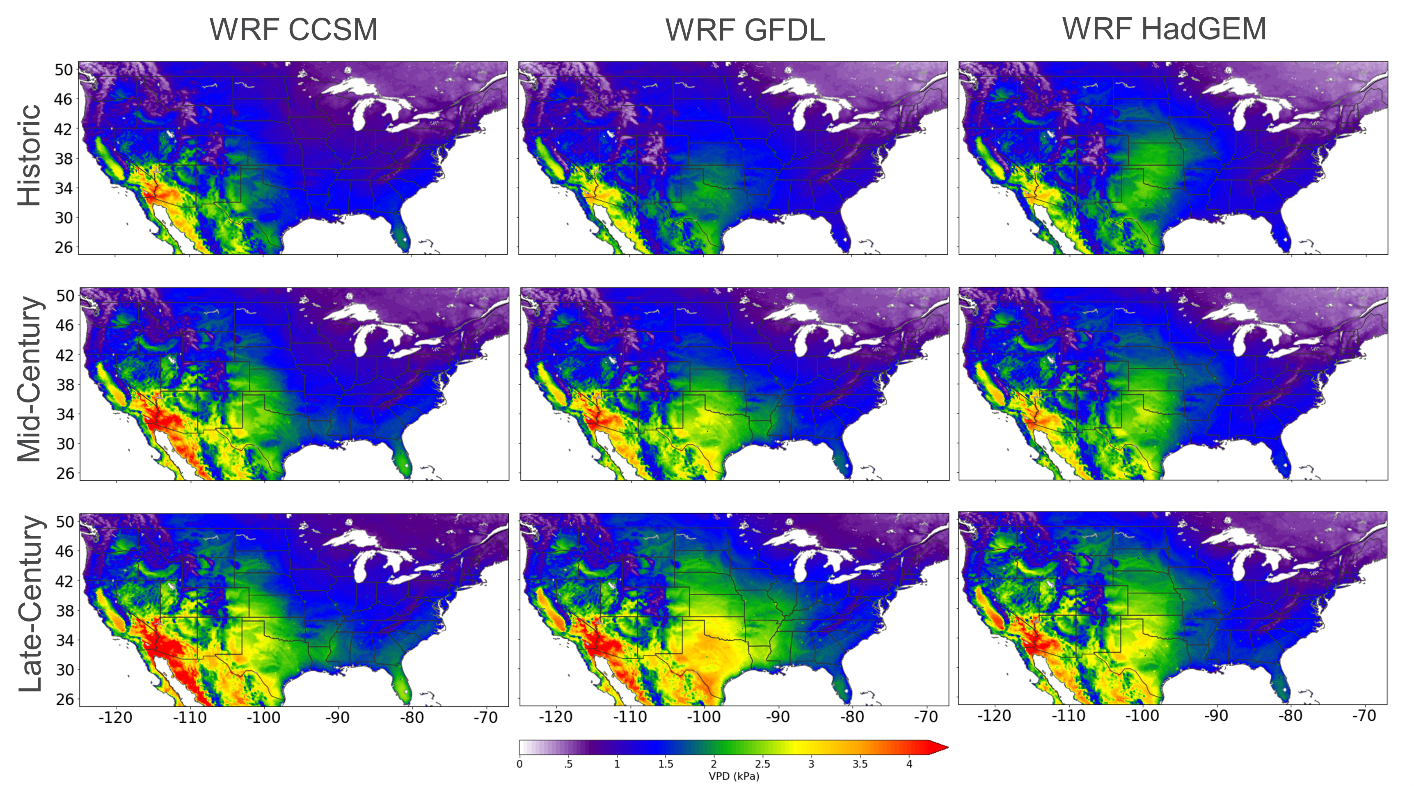


Figure S6. Spatial comparison of annual mean VPD: 1995 – 2004 (Historic), 2045 – 2054 (Mid-Century) and 2085 -2094 (Late-Century). VPD is calculated with WRF CCSM (left column), WRF GFDL (middle column) and WRF HadGEM (right column). This figure was generated using the Matplotlib^68^ library for the Python programming language (https://matplotlib.org/).


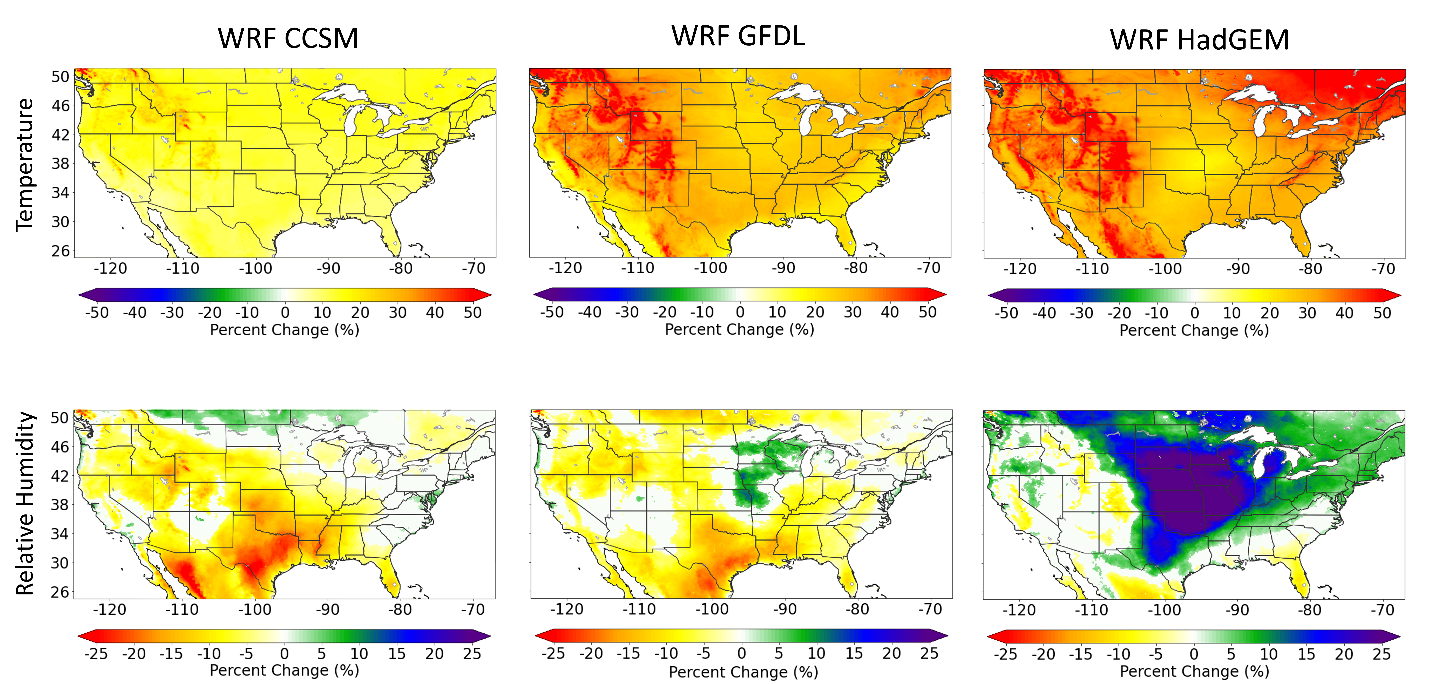


Figure S7. The percent change between historic and late-century time frames for the June, July, and August daily averaged maximum temperature (top row) and daily averaged minimum relative humidity (bottom row). Only statistically significant values (shaded) on a 5% level based on a student’s T-test are included. Note, different color bars (e.g. red temperatures values indicate warmer temperatures differences and red relative humidity values indicate drier relative humidity differences). This figure was generated using the Matplotlib^68^ library for the Python programming language (https://matplotlib.org/).


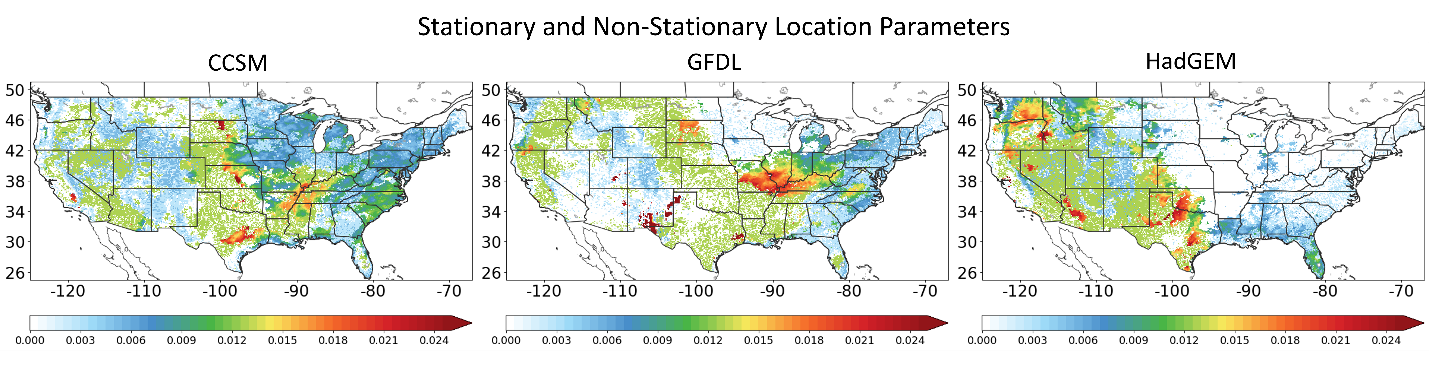


Figure S8. Stationary and linear non-stationary GEV models applied to each grid space with WRF CCSM (CCSM), WRF GFDL (GFDL) and WRF HadGEM (HadGEM). White grid spaces (location parameter = 0) in the United States indicate where a stationary GEV model was applied. Grid spaces with values greater than 0, shows the measure the location parameters increase per year in the non-stationary GEV model. This figure was generated using the Matplotlib^68^ library for the Python programming language (https://matplotlib.org/).


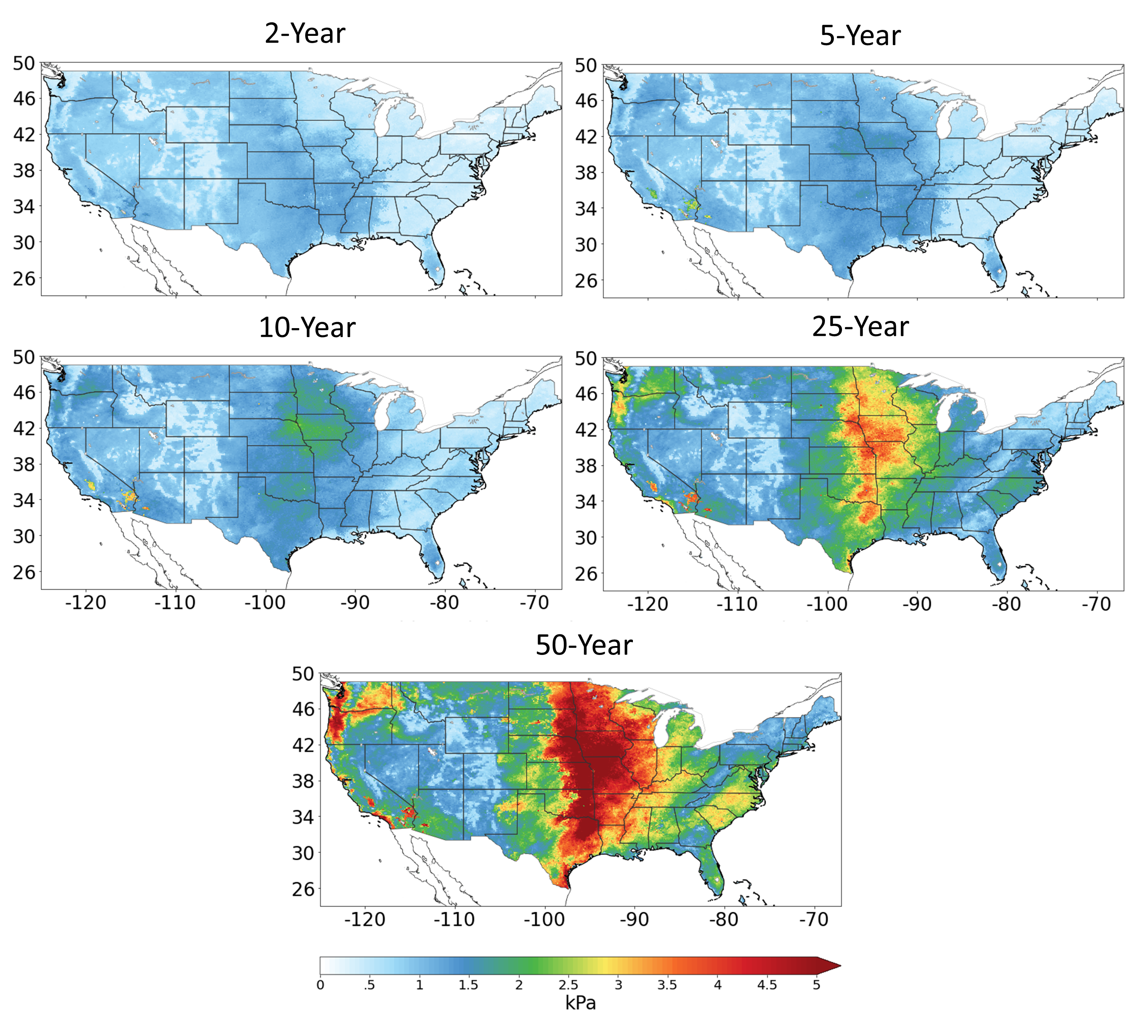


Figure S9. Ensemble model uncertainty. Differences between the ensemble 5^th^ and 95^th^ percentile for 2, 5, 10, 25 and 50-year return periods. This figure was generated using the Matplotlib^68^ library for the Python programming language (https://matplotlib.org/).
